# The model of epidemic (COVID-19) prevention and control in rural of China

**DOI:** 10.1186/s13054-020-02874-x

**Published:** 2020-04-14

**Authors:** Bao Fu, Xiaoyun Fu

**Affiliations:** Department of Critical Care Medicine, Affiliated Hospital of Zunyi Medical University, Dalian Road 149, Zunyi City, 563000 Guizhou Province China

Dear editor,

In December 2019, novel coronavirus disease 2019 (COVID-19) broke out in Wuhan and then quickly spread to various places in China [1]. The outbreak of COVID-19 coincides with the Chinese Lunar New Year. About 5 million people left Wuhan and returned to their hometown [2]. The vast majority of the five million returned to rural in China. China’s rural population is 577.61 million, and it is widely distributed in more than 691,510 villages. Therefore, the prevention and control of the epidemic in villages were facing great challenges.

The local government acted quickly and formulated some effective measures. Firstly, they checked the returnees from Hubei Province and isolated them at homes. During isolation, body temperature and symptoms were reported daily. Secondly, they minimize the flow of people. The Chinese government encouraged people to stay at home and discouraged mass gatherings. The village set up checkpoints at all intersections in the countryside to persuade the migrants to return (Fig. 1). Thirdly, they popularized the knowledge of epidemic prevention and let people know how to do well in self-protection. The unmanned aerial vehicle was used to supervise and publicize epidemic prevention knowledge. Fourthly, they fought panic with information. The government prevented people’s panic by sharing the latest information through the media. Fifthly, for COVID-19, the government implemented free medical treatment to reduce patients’ worries. This can promote patients to see a doctor as soon as possible and timely treatment, to prevent further aggravation of the condition. Sixthly, they guarantee the daily needs of the people. Seventhly, they delayed return to work and school. Schools in rural areas have also been delayed, with teachers teaching online through the Internet. Eighthly, people diagnosed with COVID-19 were isolated and treated in designated hospitals. Ninthly, they use big data to perfect tracking management. The app that can query the flights and trains that the confirmed patients have taken has also been developed. Finally, the discharged patients need to continue medical isolation and observation for half a month before they can enter the society.

**Fig. 1:**
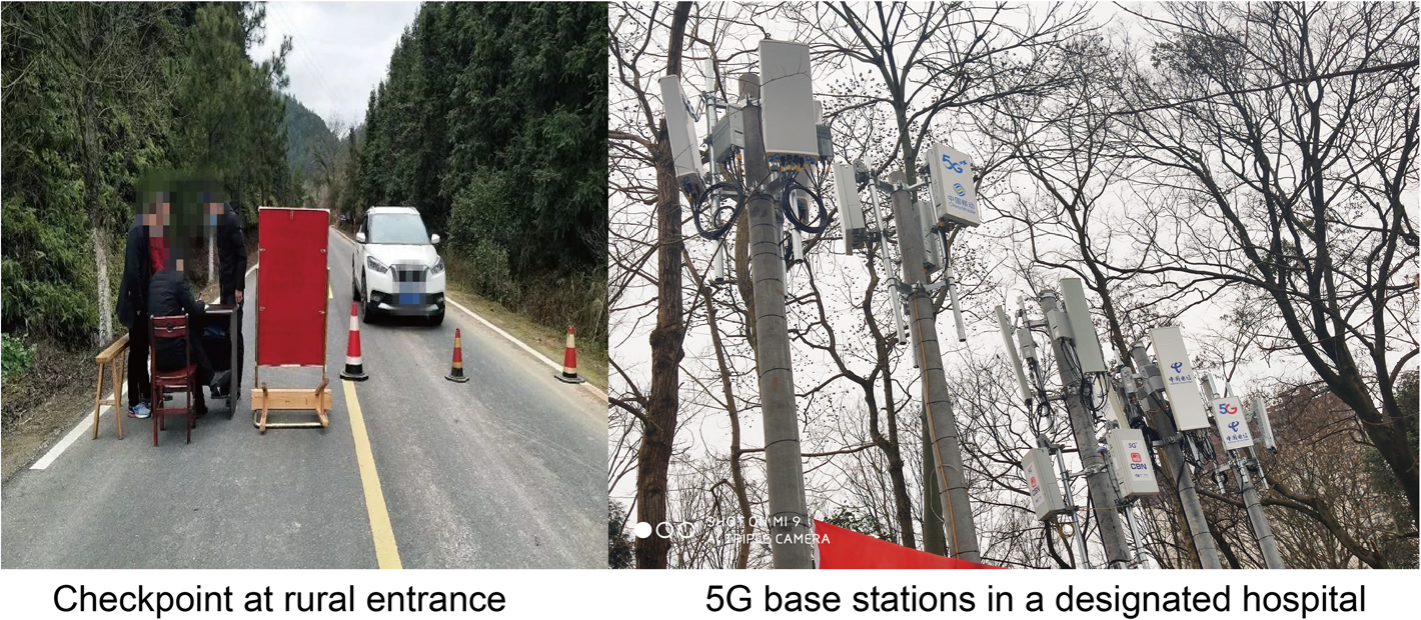
The checkpoint at rural entrance (left) and 5G base stations in a designated hospital (right)

At present, China’s epidemic prevention and control has achieved great success. This success is the result of the joint efforts of all Chinese people. We hope that China’s experience can help other countries.

## Data Availability

Not applicable.
